# Spectroscopic Ellipsometry of Plasmonic Gratings—Ideal Parameters for Sensing and Subpicometer Measurement Uncertainty

**DOI:** 10.1021/acsomega.5c00951

**Published:** 2025-04-02

**Authors:** Deshabrato Mukherjee, Sven Burger, Thomas Siefke, Jeetendra Gour, Bernd Bodermann, Peter Petrik

**Affiliations:** † Institute for Technical Physics and Materials Science, HUN-REN Centre for Energy Research, Budapest 1121, Hungary; ‡ Doctoral School on Materials Sciences and Technologies, Óbuda University, Budapest 1034, Hungary; § Zuse Institute Berlin, Berlin 14195, Germany; ∥ JCMwave GmbH, Berlin 14050, Germany; ⊥ Institute of Applied Physics, Friedrich Schiller University, Jena 07737, Germany; # Fraunhofer Institute for Applied Optics and Precision Engineering, Jena 07745, Germany; ∇ Physikalisch-Technische Bundesanstalt, Braunschweig 38116, Germany; ○ Department of Electrical Engineering, Institute of Physics, Faculty of Science and Technology, University of Debrecen, Debrecen 4032, Hungary

## Abstract

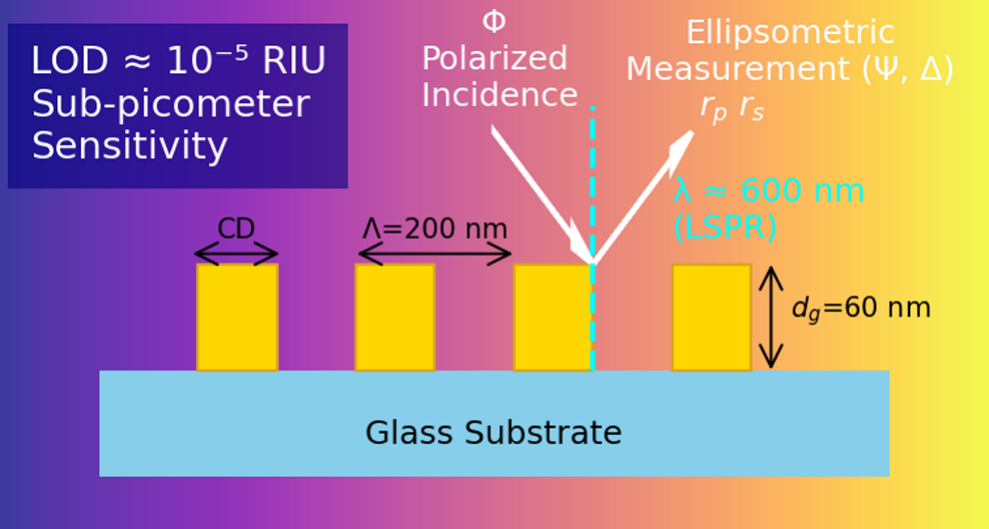

Gold gratings were measured by spectroscopic ellipsometry in reflection mode and modeled by the finite element method to investigate the capabilities of optical dimensional metrology for plasmonic diffractive structures. The gratings were prepared by electron beam lithography using parameters determined by finite element simulations for significant variations of the amplitude ratio and phase shift of the polarized reflection coefficients to achieve high sensitivity for both the measurement of the grating dimensions and the sensing capabilities. The sensitivity largely depends on the values in the five-dimensional parameter space including the grating parameters such as the critical dimension, the period, and the thickness of the grating, as well as the measurement parameters comprising the wavelength and the angle of incidence. The best limit of detection values are in the picometer range for the critical dimension and the thickness of the overlayer, and ≈10^–5^ for the refractive index.

## Introduction

Diffraction-based optical dimensional metrologies^1,2^ such as goniometric or Fourier scatterometry^3−5^ on periodic structures have been revealed to be powerful methods due to their high accuracy, speed,^6^ and nondestructive nature. The starting point of developing traceable metrology is the instrumentation^7^ and the evaluation models^8^ based on accurate standard samples^9^ as well as reference methods for verification.^10^ In the development of reference standards, materials based on silicon or its oxides have been used;^9^ however, for a range of applications, such as microelectronics^11^ and sensors,^12^ the development of the metrology of periodic diffractive plasmonic nanostructures is necessary.

Optical sensors combine high sensitivity with integrability, instrumental simplicity, and high speed.^13^ The sensing performance can be increased by achieving a resonant condition such as in the Kretschmann configuration using surface plasmon resonance.^14−16^ Although the Kretschmann arrangement is an excellent basis for versatile developments including Bragg multilayers,^17,18^ lateral scanning of reference surfaces,^19^ or combinatorial samples,^20^ cells with reduced size and simplicity can be built using local surface plasmon resonance (LSPR) by gold (or other plasmonic) nanomaterials.^21,22^

Reconstructing plasmonic surface nanostructures is challenging by optical methods including spectroscopic ellipsometry (SE) not only because of irregular shapes and size distributions but also due to the plasmonic properties.^23,24^ In terms of numerical methods employed for nanostructure characterizations, SE is usually limited to the application of the transfer matrix method, in which the dispersion is modeled by analytical functions or by tabulated data. Also, effective medium approximations (EMA) are used.^25^ Although finite-difference time-domain^26^ (FDTD), rigorous coupled wave analysis^27^ (RCWA) and finite element^22^ (FEM) methods have been demonstrated in increasing numbers,^22,26^ these applications in well-defined periodic structures on plasmonic materials have been lacking.

In this work, we investigate the dimensional metrology and sensing properties of gold grating structures designed using FEM simulations and created by electron beam lithography. We show that despite smaller imperfections the 2D FEM model represents the measured spectra well, and it is much better than EMA predictions. The sensitivity of SE measurements on the grating parameters depends very much on the experimental conditions, such as the wavelength (λ), the angle of incidence (Φ), and the parameters of the grating. It is also shown that the gratings reveal very high sensitivity for the ellipsometric measurement of the ambient or the overlayer. The grating parameters can be optimized for the used ranges of λ and Φ.

## Experimental Details

**Figure 1:**
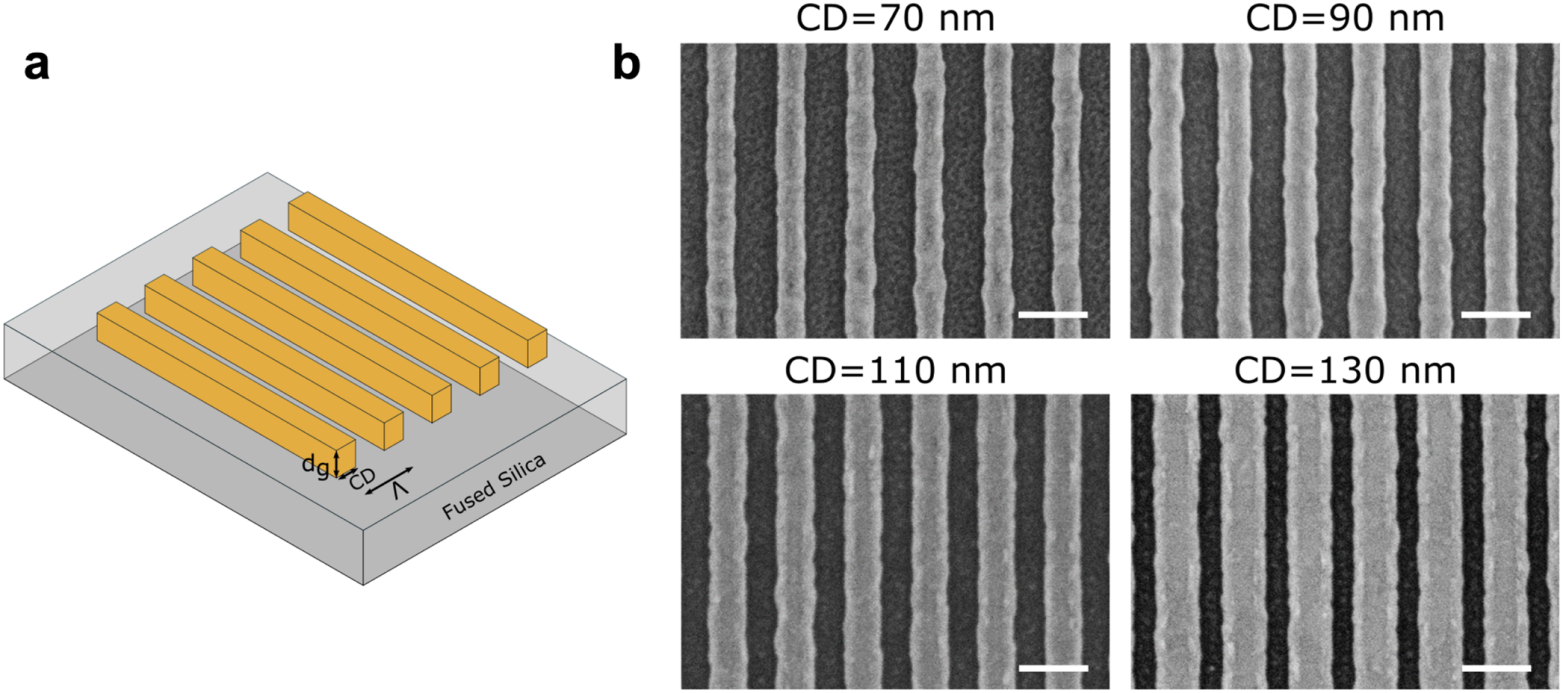
(a) A 3D schematic illustration of 1D gold gratings with parameters *d*_*g*_, CD, and Λ created on a fused silica substrate. (b) SEM images of the fabricated gold grating structures of different CDs. The length of the scale bar is 200 nm.

Gold gratings with a period of Λ = 200 nm, thickness of *d*_*g*_ = 60 nm, and line widths (CD) of 70, 90, 110, and 130 nm were prepared on glass substrates using electron beam lithography and deposition (Figure 1). Unlike the fabrication of 1*D*/2D arrays of plasmonic nanoantennas based on the liftoff technique as demonstrated in our previous work,^28−30^ the fabrication of the designed subwavelength gratings of 200 nm period and the designed critical dimensions (CDs) was not feasible due to the instability of T-shaped profile as commonly used in liftoff processes. To overcome this limitation, a top-down fabrication approach was implemented. This fabrication method shares similarities with the top-down fabrication of Si nanostructures described in ref (31).

For the sample fabrication, a fused silica wafer with a diameter of approximately 150 mm was used as the substrate. The wafer was first cleaned in an automatic wafer cleaner (SB30 OPTIwet) with Caro’s acid, mega-sonic agitated water, and high-pressure ammonia. A gold layer with a thickness of 60 nm, preceded by a 3 nm chromium (Cr) adhesion layer, was deposited onto the cleaned substrate using electron beam evaporation (V700, JEVATEC GmbH). Subsequently, using Ionfab 300-Oxford Instruments, a 10 nm Cr layer was sputtered onto the gold surface to serve as a hard mask for subsequent etching processes.

Afterward, the samples are coated with an HMDS adhesion promoter in a Sawatec 200/300 for 60 s at 110 °C after 10 min of dehydration at 150 °C.^30^ Immediately following the HMDS process, a 100 nm thick negative-tone resist OBER-CAN038 AE 2.0CP (Tokyo Ohka Kogyo Co., Ltd.) was spin-coated onto the Cr hard mask and patterned via electron beam lithography using a Vistec 350OS electron beam writer to define the designed grating structures. The patterned resist served as a mask for transferring the grating design into the Cr layer using ion beam etching (IONFAB 300). Following this, the resist mask was removed by etching in an oxygen plasma (500 W, 2 min) to minimize redepositing on the grating sidewalls.

The grating structure, now transferred into the Cr hard mask, was subsequently etched into the underlying gold layer using a reactive ion beam etching process (IONFAB 300) optimized for the slow etch rate of gold. This process resulted in the formation of gold grating structures. At this stage, a residual thin Cr layer remained on the structures and was removed using a dry etching process (Cr-ICP) with chlorine-based chemistry using Oxford Instruments Plasma Pro 100. Finally, the fabricated wafer was diced into chips of the desired sizes for subsequent optical experiments.

The dimensional parameters of the gratings were designed using calculations by the JCMsuite 6.0.10 finite element solver aiming to maximize the variation of the amplitude ratio (Ψ = tan^–1^(|*r*_*p*_/*r*_*s*_|)) and phase shift (Δ = arg(*r*_*p*_/*r*_*s*_)) of the reflection coefficients of light polarized parallel (*r*_*p*_) and perpendicular (*r*_*s*_) to the plane of incidence. The respective measurements on these samples were performed using a Woollam M-2000DI ellipsometer at Φ = 60° to 75°. The limit of detection^32^ (LOD) was calculated using a Python interface with the JCMsuite package, the details of which is described in the next section.

## Results

**Figure 2:**
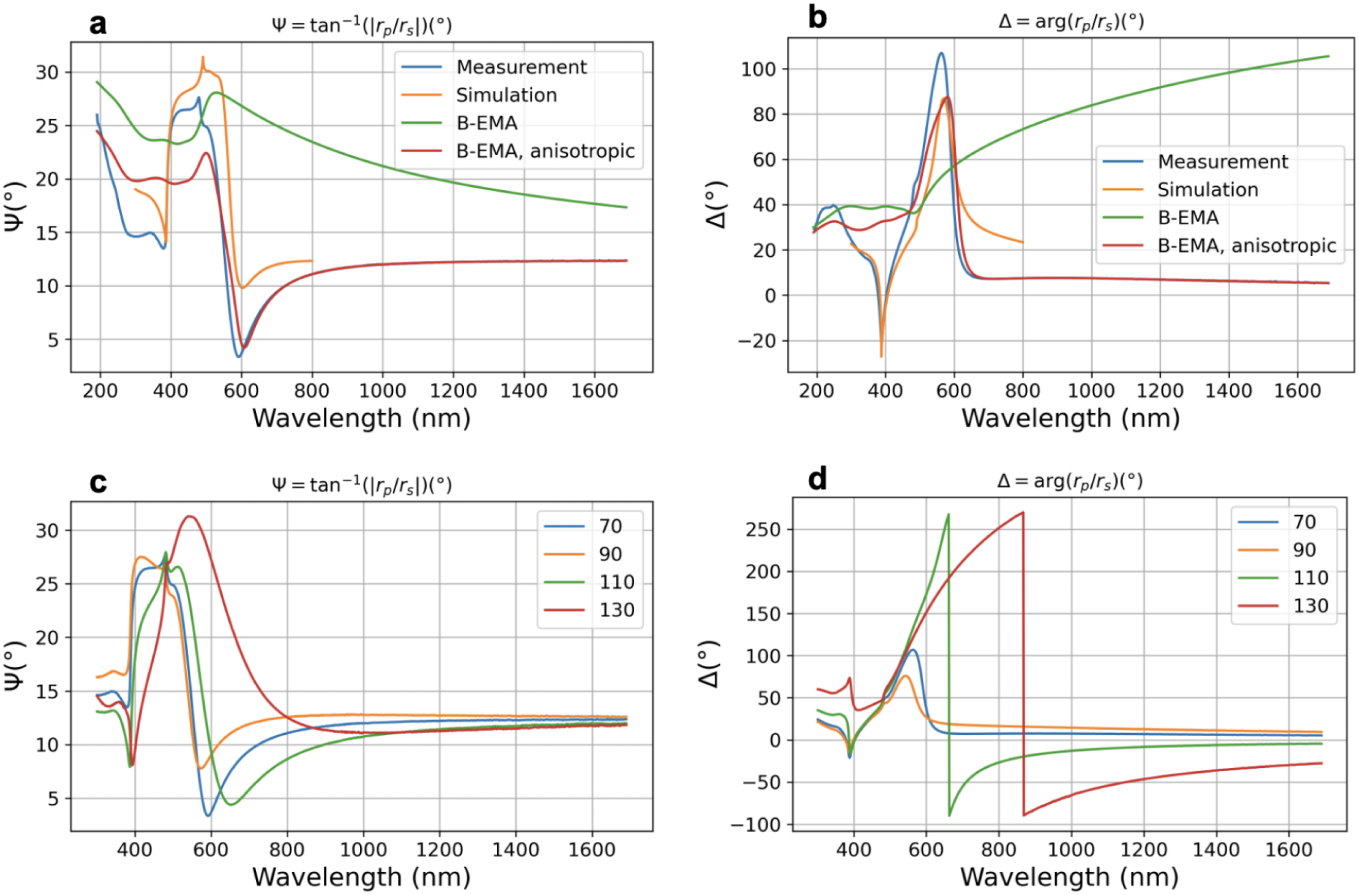
(a) and (b) show measured (plane of incidence perpendicular to the grating lines) and FEM-simulated Ψ and Δ spectra at Φ = 70°, CD = 70 nm, and *d*_*g*_ = 60 nm. The standard isotropic BEMA model is plotted using *d*_*g*_ = 60 nm, and the volume fraction of 0.65, corresponds to CD = 70 nm, and Λ = 200 nm. In the BEMA simulation, the rotation angles and the depolarization parameters were fitted. (c) and (d) represent the measured Ψ and Δ spectra for the prepared grating samples with different values of CD in nanometers, as well as Φ = 70° and *d*_*g*_ = 60 nm.

Figure 2 shows the measured and calculated Ψ and Δ spectra. The FEM calculation assumes a perfect grating using the nominal parameters in a 2D model. The figure also shows spectra calculated using the EMA.^33^ This model assumes that the gold grating is a layer with a refractive index that combines the refractive index of gold and air. The most important requirement for the EMA model’s validity is that the features’ size is smaller than the wavelength of illumination (quasi-static limit).^34^ The revealed limiting values of the wavelength and feature size ratio (λ/Λ) range between 0.1^34^ and 0.3.^35,36^ The EMA calculations were performed using isotropic and anisotropic Bruggeman (BEMA) models revealing that the lack of taking into account anisotropy is the major cause of the failure of general BEMA calculations. The speed of the EMA calculation allows the fitting of the spectra as also shown in Figure 2. The deviation between the anisotropic BEMA model and the measurement starts to increase at λ = 600 nm (Figure 2a,b), supporting a value of λ/Λ = 0.3.

**Figure 3:**
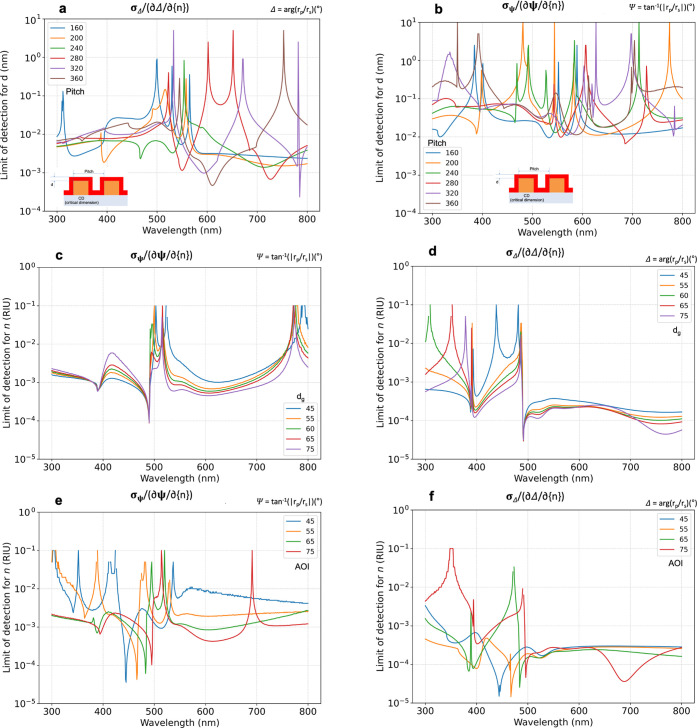
LOD graphs of *d* for *d*_*g*_ = 60 nm, CD = 110 nm, and different Λ values (a,b), *n*_*a*_ for Λ = 200 nm, CD = 110 nm, Φ = 70° and different *d*_*g*_ values (c d) as well as *n*_*a*_ for Λ = 200 nm, CD = 110 nm, *d*_*g*_ = 60 nm and different Φ values (e,f) calculated from (a,c,e) Ψ and (b,d,f) Δ.

The uncertainty of a measured value can be determined from a statistical analysis (standard deviation) of repeated measurements. The sensitivity of the measurement of parameters of a model that describes a physical system can be determined using the concept of LOD.^32^ In this method sensitive parameters are identified by analyzing the  and  derivatives, where *P* denotes the individual parameters. Knowing the standard deviation (σ_Ψ,Δ_) of the measured Ψ and Δ values, the LOD can be estimated using , as shown in Figure 3 for *P* = *d* and *P* = *n*_*a*_, where *d* is the thickness of the overlayer to be measured having a dispersion of *n* = 1.45 + 0.01/λ^2^ (λ in micrometers) and *n*_*a*_ is the refractive index of the ambient in refractive index units (RIU).

**Figure 4:**
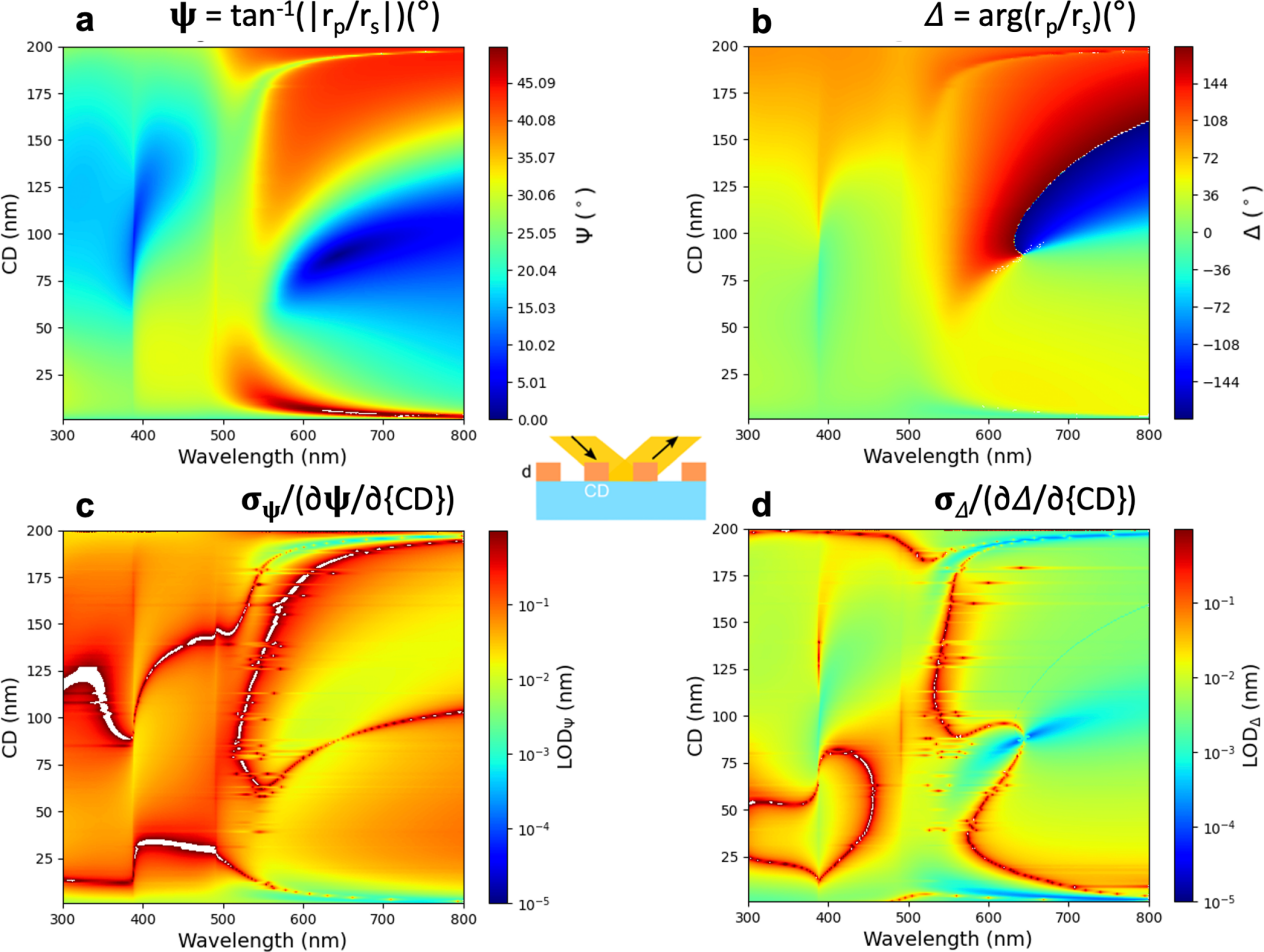
Maps of (a) Ψ and (b) Δ in the λ-CD plane for Λ = 200 nm, Φ = 70°, and *d*_*g*_ = 60 nm. Maps of (c) LOD_Ψ_(CD) and (d) LOD_Δ_(CD) calculated from (a) and (b), respectively. The *z*-range is stretched (not autoscaled) in (c) and (d) for better visibility, resulting in white areas for the cut values.

**Figure 5:**
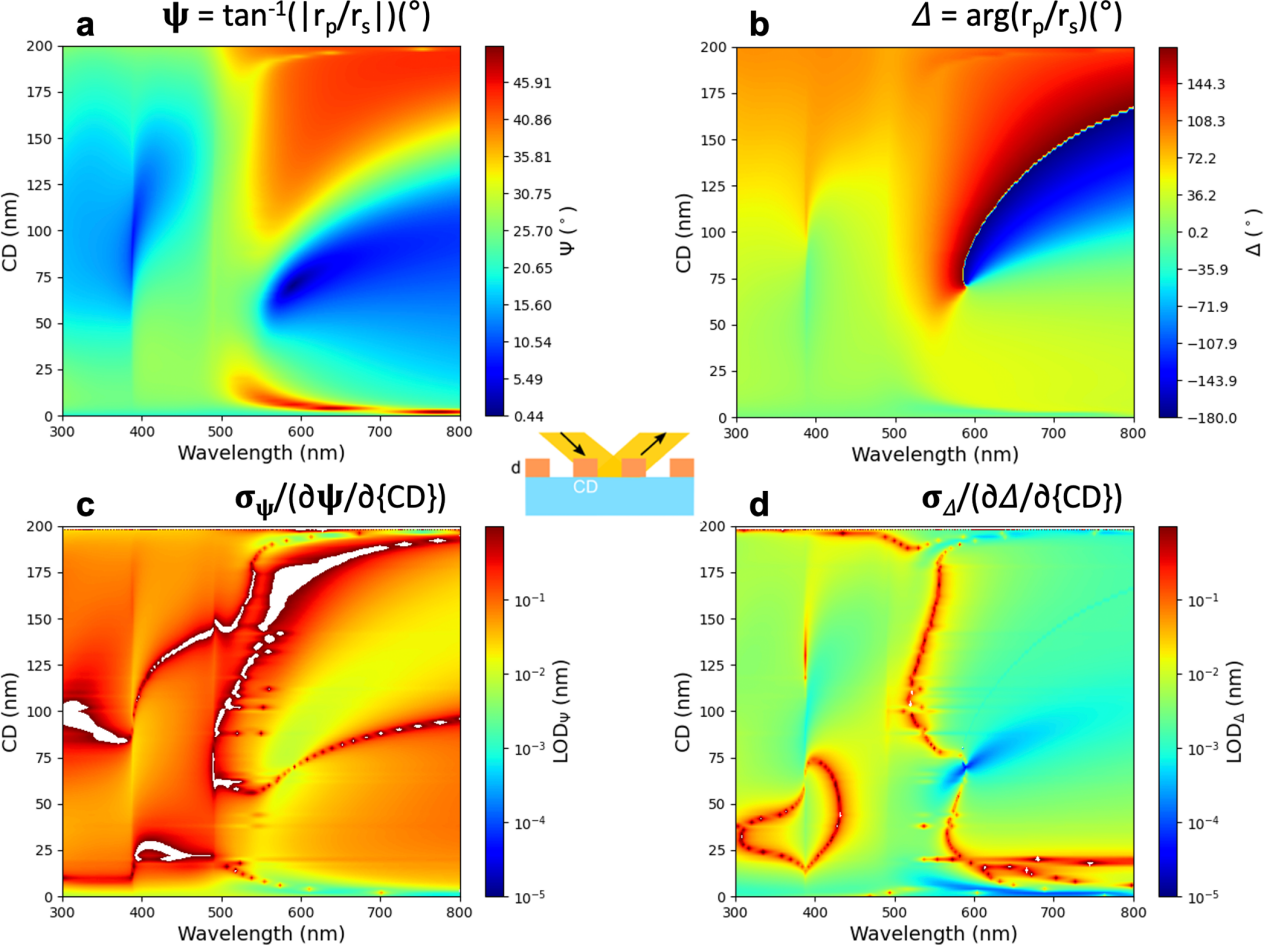
Maps of (a) Ψ and (b) Δ in the λ-CD plane for Λ = 200 nm, Φ = 70°, and *d*_*g*_ = 45 nm. Maps of (c) LOD_Ψ_(CD) and (d) LOD_Δ_(CD) calculated from (a) and (b), respectively. The *z*-range is stretched (not autoscaled) in (c) and (d) for better visibility, resulting in white areas for the cut values.

Since the dimensional parameters of the grating are also part of the model, the sensitivity for the measurement of those parameters (Λ, CD, *d*_*g*_) can also be tested using the same approach as *n*_*a*_ and *d*_*g*_. In our five-dimensional parameter space that includes the three grating parameters (Λ, CD, *d*_*g*_) and two measurement parameters (Φ and λ), 2D projections can be generated for arbitrary pairs of parameters, as shown in Figures 4 and 5 for CD and λ. The LOD maps which are shown in both sets of Figures 4c,d and 5c,d are proportional to the reciprocal values of the derivatives of maps in Figures 4a,b and 5a,b respectively along the CD axis revealing subpicometer sensitivity in certain regions of the Δ map in Figures 4d and 5d – see the dark blue region around CD = 80 nm and λ = 620 nm in Figure 4d and a more prominent effect for at around CD = 70 nm and λ = 590 nm in Figure 5d. Note that the λ value corresponds to the resonance wavelength of localized plasmons in gold.^16^

## Discussion

Although the measured and nominal Ψ and Δ spectra in Figure 2 are very similar, they only show a qualitative agreement, which cannot be significantly improved by fitting the model parameters. There are multiple candidates for the explanation including the nonideal structure of the grating (see Figure 1, revealing, e.g., a possible line-edge roughness), and the possible need for a 3D FEM model, to be investigated in the following study. The basic EMA approaches also fail to describe the measurement because of disregarding mirror charges, anisotropy, and other effects.^23,25^ Note, however, that the grating can be fitted using anisotropic EMA in the quasistatic limit, i.e., for wavelengths above 600 nm, as discussed above.

The analysis of the five-dimensional parameter space (λ, Φ, CD, *d*_*g*_, and Λ) in FEM provides the opportunity to investigate sensitivities for both dimensional metrology and sensing performance. The fine-structured values of the parameter space (see e.g., a projection of Ψ and Δ values to the parameter plane defined by λ and CD for fixed values of the other parameters in Figures 4a, 5a and 4b, 5b) combined with the limited speed of FEM calculations makes it difficult to find optimal sets of parameters in terms of criteria, such as the sensitivity for dimensional measurements or the sensing performance discussed here. In future works we plan to exploit automatic differentiation for obtaining parameter sensitivities.^37^ In this work we only demonstrate LOD calculations for subsets of parameters corresponding to the values of the manufactured gratings, the dimensions of which were determined by the limitations of production (e.g., electron lithography) and preliminary calculations.^38^

Calculating the LOD_*n*_ of the refractive index of the ambient does not require a new optical model, only the derivatives of the measured Ψ and Δ values must be calculated for small (0.01) changes of *n*_*a*_, as shown in Figures 3c–f. In both cases of comparison with respect to *d*_*g*_ and Φ, the dips of best LOD values are located between λ = 400 nm and λ = 500 nm for both  and , reaching values as low as  and . The dip position is slightly shifted to larger wavelengths with increasing *d*_*g*_ and Φ. There are numerous low-sensitivity peaks for both  and , but significantly more for the former. Although in a tough’competition’, the best overall LOD_*n*_ value was identified for *d*_*g*_= 55 nm from . Because the sensitivities are not very different, the thickness of the grating can also be used to adjust the wavelength for the best sensitivity. In the case of Φ, similar patterns are observed for  where additional dips appear slightly below the wavelengths of 400 and 700 nm for Φ = 60° and Φ = 75°, respectively.

Calculating LOD_*d*_ for the thickness of the overlayer requires a new model that adds a uniform layer^22^ to the grating structure (see the insets in Figure 3a,b). The difference between the  values calculated from  and  are more than an order of magnitude in favor of , similar to the case of LOD_*n*_. The sensitivities are in the picometer range. Using *n* ≈ 1.45 for a protein layer the estimated LOD of surface mass density is ≈10^–9^ g/cm^2^ (10 pg/mm^2^) using De Feijter’s formula^39^ of Γ = (*nd*)/*a*, where *a* = ∂*n*/∂*c* = 0.18 cm^3^/g, and *c* stands for the concentration in g/cm^3^. In this approach, the thickness and refractive index sensitivity are converted to mass sensitivity taking advantage of the fact that the optical measurement is sensitive to the product of *n* · *d*, even if they cannot be separated due to parameter correlations for ultrathin layers. There are only a few peak regions for both  and , where the LOD_*d*_ value increases above 1 nm. There are high sensitivity dips for both  and  depending on Λ, but the majority of the values are between  and  nm for Ψ and  to  nm for Δ for the majority of the Λ values.

Similar to the sensing performance, the sensitivities or LOD values for dimensional metrology depend greatly on the location in the five-dimensional parameter space. Figures 4 and 5 illustrate the fine structure of the Ψ and Δ surfaces in the λ - CD plane for fixed values of Λ, Φ, and *d*, revealing large gradients and distinct resonance features in both parameters. These resonances correspond to optical modes excited in the system, with sharp transitions indicating strong optical field confinement and interaction with the structured geometry. When *d*_*g*_ = 45 nm (Figure 5a,b), the Ψ and Δ maps display steep phase and amplitude variations, particularly in regions of strong optical coupling. As *d*_*g*_ increases to 60 nm (Figures 4a,b), the LOD landscape shifts, reflecting the impact of feature size on optical interactions. We show maps of  (Figures 4c and 5c) and  (Figures 4d and 5d), but LOD values can be calculated from the partial derivatives in each point of the parameter space for each of the five parameters. Both  and  span more than 5 orders of magnitude, demonstrating extremely high sensitivity contrast across the spectrum. Complementing the results shown in Figure 3 for sensing performance, the LOD values from Δ are also better by at least an order of magnitude than those from Ψ. This is expected since Δ is more sensitive to phase change, and phase shifts are typically more responsive to small structural variations than intensity-based Ψ measurements. For *d*_*g*_ = 45 nm, the typical  values are around ≈10^–1^ to 10^–2^ nm, whereas for *d*_*g*_ = 60 nm, the LOD landscape is slightly modified, reflecting a shift in the optimal sensitivity regions due to the increased feature size. There were also two locations of significantly better LOD values emerging in both cases which can be attributed to strong resonant interactions. For *d*_*g*_ = 45 nm, one of the optimal sensitivity regions appears at around λ = 600 nm (LSPR wavelength of gold) and CD = 80 nm, whereas, at *d*_*g*_ = 60 nm, this resonance shifts to CD = 90 nm and appears broadened, indicating a more distributed plasmonic coupling effect due to the increased feature size. The second optimal region, shifted by ≈ 200 nm (with Λ = 200 nm) to λ ≈ 400 nm, is present in both cases. However, at *d*_*g*_ = 60 nm, the LOD minima appear more dispersed, suggesting a modified diffractive interaction due to the increased CD value. The modifications observed between *d*_*g*_ = 45 nm and *d*_*g*_ = 60 nm emphasize the strong dependence of LOD values on nanoscale geometry, reinforcing the need for advanced modeling and experimental validation in high-precision optical metrology.

## Conclusions

2D FEM models were developed to calculate the SE response of gold gratings. The calculations show a good agreement with SE measurements on gratings prepared by electron beam lithography. The discrepancy between the measured and calculated spectra cannot be eliminated by parameter fitting which indicates imperfections (line edge roughness, side-wall angle, etc.) in the grating or limitations of the 2D models for the description of the structures. LOD values calculated from the FEM models were orders of magnitude better for the phase-sensitive SE parameter (Δ) revealing values in the picometer range for the thickness of the overlayer and the CD parameter, and 10^–5^ for the refractive index. Besides the high sensitivities in non-Kretschmann configurations, we have shown the importance of the optimization of both the grating and measurement parameters for best sensitivities. To reveal the sensitivity values by measurements on the gratings is planned in the next publication.
